# Supplementary material for: The causal relationship between the human gut microbiota and pyogenic arthritis: a Mendelian randomization study
Source: Front Cell Infect Microbiol. 2024 Nov 26;14:1452480. doi: 10.3389/fcimb.2024.1452480 (PMC11629706; doi:10.3389/fcimb.2024.1452480)
Supplement: Supplementary file 1 [file DataSheet1.zip › mendelian test/ebi-a-GCST90027709.csv_leave_one_out.pdf]

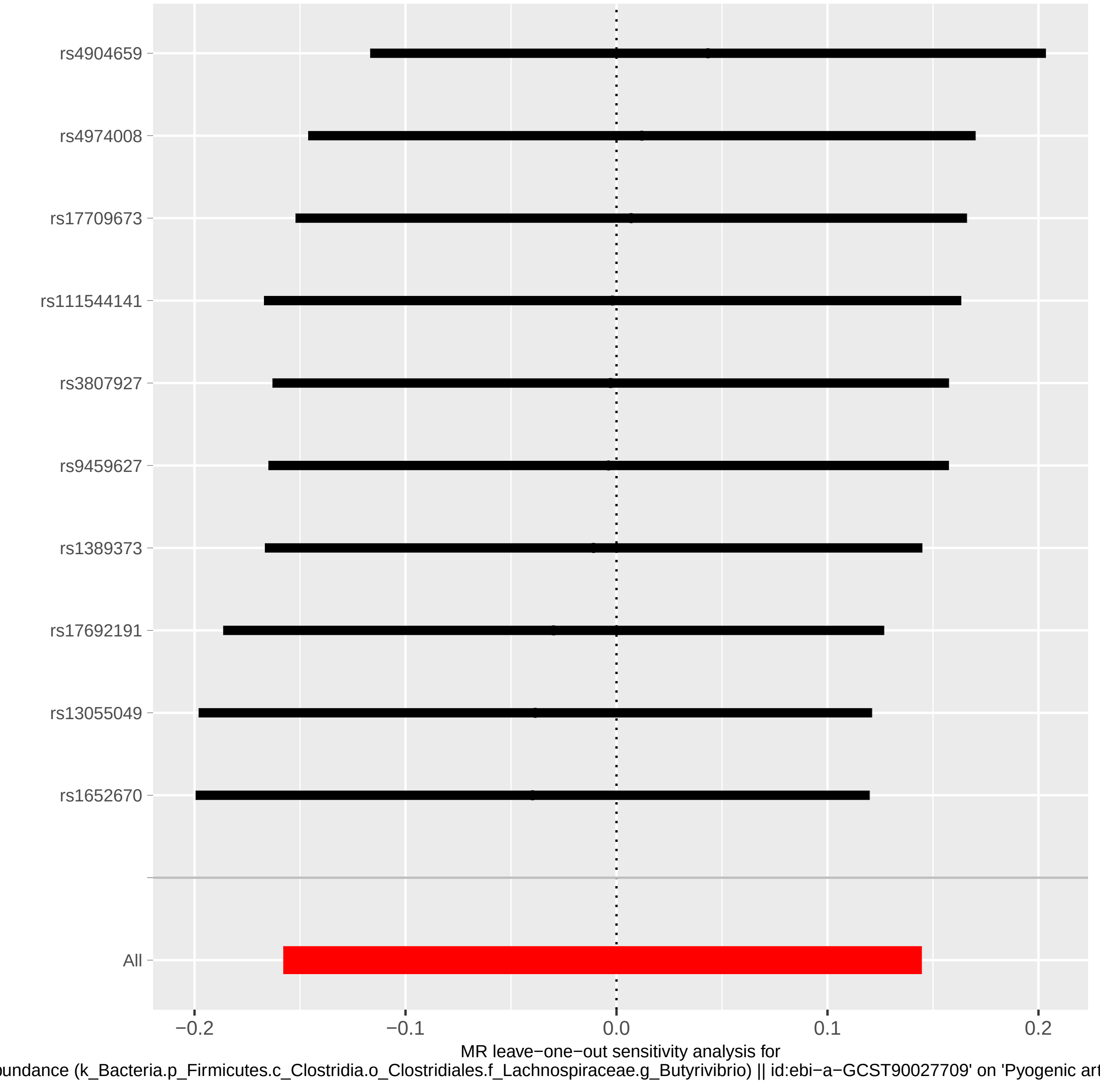
rs4904659
rs4974008
rs17709673
rs111544141
rs3807927
rs9459627
rs1389373
rs17692191
rs13055049
rs1652670
All
−0.2
−0.1
0.0
0.1
0.2
MR leave−one−out sensitivity analysis for
undance (k_Bacteria.p_Firmicutes.c_Clostridia.o_Clostridiales.f_Lachnospiraceae.g_Butyrivibrio) || id:ebi−a−GCST90027709' on 'Pyogenic art
